# Artificial selection footprints in indigenous and commercial chicken genomes

**DOI:** 10.1186/s12864-024-10291-5

**Published:** 2024-04-30

**Authors:** Siwen Wu, Tengfei Dou, Kun Wang, Sisi Yuan, Shixiong Yan, Zhiqiang Xu, Yong Liu, Zonghui Jian, Jingying Zhao, Rouhan Zhao, Hao Wu, Dahai Gu, Lixian Liu, Qihua Li, Dong-Dong Wu, Changrong Ge, Zhengchang Su, Junjing Jia

**Affiliations:** 1 Faculty of Animal Science and Technology, Yunnan Agricultural University, Kunming, Yunnan China; 2 Department of Bioinformatics and Genomics, The University of North Carolina at Charlotte, Charlotte, NC 28223 USA; 3 State Key Laboratory of Genetic Resources and Evolution, Kunming Institute of Zoology, Chinese Academy of Sciences, Kunming, China; 4 Center for Excellence in Animal Evolution and Genetics, Chinese Academy of Sciences, Kunming, China

**Keywords:** Chicken, Domestication, Evolution, Selective sweep, SNPs, Permutation

## Abstract

**Background:**

Although many studies have been done to reveal artificial selection signatures in commercial and indigenous chickens, a limited number of genes have been linked to specific traits. To identify more trait-related artificial selection signatures and genes, we re-sequenced a total of 85 individuals of five indigenous chicken breeds with distinct traits from Yunnan Province, China.

**Results:**

We found 30 million non-redundant single nucleotide variants and small indels (< 50 bp) in the indigenous chickens, of which 10 million were not seen in 60 broilers, 56 layers and 35 red jungle fowls (RJFs) that we compared with. The variants in each breed are enriched in non-coding regions, while those in coding regions are largely tolerant, suggesting that most variants might affect *cis*-regulatory sequences. Based on 27 million bi-allelic single nucleotide polymorphisms identified in the chickens, we found numerous selective sweeps and affected genes in each indigenous chicken breed and substantially larger numbers of selective sweeps and affected genes in the broilers and layers than previously reported using a rigorous statistical model. Consistent with the locations of the variants, the vast majority (~ 98.3%) of the identified selective sweeps overlap known quantitative trait loci (QTLs). Meanwhile, 74.2% known QTLs overlap our identified selective sweeps. We confirmed most of previously identified trait-related genes and identified many novel ones, some of which might be related to body size and high egg production traits. Using RT-qPCR, we validated differential expression of eight genes (*GHR, GHRHR, IGF2BP1, OVALX, ELF2, MGARP, NOCT, SLC25A15*) that might be related to body size and high egg production traits in relevant tissues of relevant breeds.

**Conclusion:**

We identify 30 million single nucleotide variants and small indels in the five indigenous chicken breeds, 10 million of which are novel. We predict substantially more selective sweeps and affected genes than previously reported in both indigenous and commercial breeds. These variants and affected genes are good candidates for further experimental investigations of genotype-phenotype relationships and practical applications in chicken breeding programs.

**Supplementary Information:**

The online version contains supplementary material available at 10.1186/s12864-024-10291-5.

## Background

Chicken (*Gallus gallus*) has been domesticated by human for about 8000 years [1], and multiple lines of evidence show that red jungle fowl (RJF) is the major ancestor of domestic chicken all over the world [1–3]. Artificial selection has resulted in numerous chicken breeds with distinct traits in different parts of the world for various purposes, including meat and egg production as well as recreation and ornament. Particularly, intensive systematic artificial selections carried out in a few companies in the last decays have led to highly production-efficient commercial broiler and layer lines used all over the world. Understanding the genetic basis of distinct traits of traditionally bred indigenous chicken as well as of commercial broilers and layers is crucial to guide breeding programs to further improve the chicken welfare [4]. Besides commercial lines, indigenous chicken breeds are also excellent model systems to study the relationships between genotypes and phenotypes [5]. Indeed, many studies have been done to reveal artificial selection signatures on commercial broilers and layers [6–8] as well as on indigenous chickens [9–17]. These studies have identified genes or quantitative trait loci (QTLs) related to specific traits such as body size [10, 11, 18–23], meat quality [24], egg production [25], feathering [26–33], plumage color [34–37], skin color [38], behavior [39], immunity [40], crest shapes [41], bone traits [42–44], rumpless trait [45–48], and polydactyly [49, 50]. Yunnan, a southwest province of China, is one of the major centers where domestic chickens arise [3], and numerous chicken breeds have been raised in mountainous areas there. Among these indigenous chicken breeds are Daweishan, Hu, Piao, Wuding and Nine-claw chicken, each with distinct traits. Specifically, Daweishan chickens have a miniature body size (0.5 ~ 0.8 kg for female and 0.8 ~ 1.2 kg for male adults); Hu chickens have a large body size (3 kg for female and 6 kg for male adults) with extraordinarily stout legs; Piao chickens have a short tail (a rumpless phenotype); Wuding chickens have a relatively large body size with colorful feathers and thick fat; and Nine-claw chickens have nine claws with a middle body size.

To understand the domestication and genetic basis of the distinct traits of these indigenous chickens, we re-sequenced 25 Daweishan chickens, 10 Hu chickens, 23 Piao chickens, 23 Wuding chickens and four Nine-claw chickens. By comparing the single nucleotide polymorphisms (SNPs) of these indigenous chicken populations with those of 35 RJF individuals as well as of 60 broiler individuals and 56 layer individuals using a rigorous statistic model [6, 51], we were able to find numerous artificial selection signatures in the indigenous chickens, and substantially larger numbers of artificial selection signatures in broilers and layers than previously reported [6, 52, 53]. By comparing the selection signatures between the indigenous chicken breeds, RJF, broilers and layers, we found numerous genomic regions and genes related to the breed-specific traits.

## Methods

### Re-sequencing short reads from NCBI SRA

We downloaded genomic short reads of two broiler lines from NCBI Sequence Read Archive (SRA): “Broiler A” (*n* = 40, access number PRJEB15276) and “Broiler B” (*n* = 20, access number PRJEB30270). Broiler A was originally from France, and Broiler B was from the company Indian River International. We downloaded DNA short reads of three layer lines from NCBI SRA: “Layer A” (*n* = 25, access number PRJEB15189) were white egg layers, “Layer B” (*n* = 25, access number PRJEB30270) were brown egg layers, and “Layer C” (*n* = 6, access number PRJEB30270) were crossbred layers. We downloaded genomic short reads of two RJF populations from NCBI SRA: “RJF A” (n = 25, access number PRJEB30270) were from northern Thailand, and “RJF B” (*n* = 10, access number PRJEB30270) were from India.

### Re-sequencing of indigenous chicken samples

We re-sequenced 85 indigenous chicken individuals from the Experimental Breeding Chicken Farm of the Yunnan Agricultural University (Yunnan, China), including 25 Daweishan chickens aged 10 months (nine males, 16 females), 10 Hu chickens aged 7 months (five males, five females), 23 Piao chickens aged 10 months (11 males, 12 females), 23 Wuding chickens aged 10 months (11 males, 12 females) and four Nine-claw chickens aged 10 months (two males, two females).

### Short-reads DNA sequencing

Two milliliters of blood were drawn from the wing vein of each chicken in a centrifuge tube containing anticoagulant (EDTA-2 K) and stored at − 80 °C until use. Genomic DNA (10 μg) in each blood sample was extracted using a DNA extraction kit (DP326, TIANGEN Biotech, Beijing, China) and fragmented using a Bioruptor Pico System (Diagenode, Belgium). DNA fragments around 350 bp were selected using SPRI beads (Beckman Coulter, IN, USA). DNA-sequencing libraries were prepared using Illumina TruSeq® DNA Library Prep Kits (Illumina, CA, USA) following the vendor’s instructions. The libraries were subject to 150 cycles paired-end sequencing on an Illumina Novaseq 6000 platform (Illumina, CA, USA) at ~30X coverage.

### Real-time quantitative PCR (RT-qPCR) analysis

One to 2 grams of relevant tissues were collected from individual chickens of relevant breeds in a centrifuge tube and immediately frozen in liquid nitrogen, then stored at − 80 °C until use. Total RNA from each tissue sample were extracted using TRlzol reagents (TIANGEN Biotech, Beijing China) according to the manufacturer’s instructions. RT-qPCR was performed using the Bio-Rad CFX96 real-time PCR platform (Bio-Rad Laboratories. lnc, America) and SYBR Green master mix (iQTM SYBRGreen® Supermix, Dalian TaKaRa Biotechnology Co. Ltd. Add). The primers of the eight genes are listed in Supplementary Note 1. The β-actin gene was used as a reference. Primers were commercially synthesized (Shanghai Shenggong Biochemistry Company P.R.C). Each PCR reaction was performed in 25 μl volumes containing 12.5 μl of iQ™ SYBR Green Supermix, 0.5 μl (10 mM) of each primer, and 1 μl of cDNA. Amplification and detection of products was performed with the following cycle profile: 1 cycle of 95 °C for 2 min, and 40 cycles of 95 °C for 15 s, annealing temperature for 30 s, and 72 °C for 30 s, followed by a final cycle of 72 °C for 10 min. The specificity of the amplification product was verified by electrophoresis on a 0.8% agarose gel and DNA sequencing. The 2^−ΔCt^ method was used to analyze mRNA abundance. All tissues were analyzed with at least three biological replicates and each biological replicate with five technical replicates, and the means and standard deviation of all these measurements were presented in the relevant figures.

### Variant calling

We mapped the short reads of each individual chicken to the reference genome (GRCg7b) using BWA (0.7.17) [54] and SAMtools (1.9) [55] with the default settings and called variants for each individual using GATK-HaplotypeCaller (4.1.6) [56] with the default settings. After generating the GVCF files for each individual, we computed allele frequencies in the same chicken breed using the GATK-CombineGVCFs (4.1.6) tool [56]. For each chicken breed, we removed variants with Quality by depth (QD) < 2, Fisher strand (FS) > 60, Root mean square mapping quality (MQ) < 40, Strand odd ratio (SOR) > 3, Rank Sum Test for mapping qualities (MQRankSum) < − 12.5 and Rank Sum Test for site position within reads (ReadPosRankSum) < − 8 for SNPs and QD < 2, FS > 200, SOR > 10, Likelihood-based test for the consanguinity among samples (InbreedingCoeff) < − 0.8 and ReadPosRankSum < − 20 for indels.

### Genetic linkage disequilibrium (LD) analysis

LD analysis in each chicken population was performed using PopLDdecay [57] with the default settings based on the autosome SNPs called in each chicken individual of the 12 chicken populations.

### Runs of homozygosity (ROH) analysis

ROH analysis in each chicken population was done using BCFtools (1.10) [58] with the default settings based on the autosome SNPs called in each chicken individual of the 12 chicken populations. For convenience of discussion, we define a LD decay rate as the average distance for LD to decay until R^2^ = 0.2.

### Principal component analysis (PCA)

PCA was performed using PLINK (1.90) [59] with the default settings based on the autosome SNPs called in each chicken individual of the eight breeds.

### Population structure analysis

Population structure was inferred using ADMIXTURE (1.3.0) [60] with K = 2, 3, …, 12 using the default settings based on the autosome SNPs called in each chicken individual of the 12 chicken populations.

### Functional annotation of variants

We used the package ANNOVAR [61] to annotate the variants according to their locations on the reference genome into seven categories including 1) intergenic regions, 2) intronic regions, 3) coding regions (synonymous, nonsynonymous, stop gain and stop loss), 4) up/downstream of a gene, 5) splicing sites, 6) 5′ untranslated regions (5’UTRs) and 3′ UTRs, and 7) non-coding RNA regions. We used the tool Ensembl Variant Effect Predictor (VEP) [62] to predict the impact of amino acid-altering SNPs.

### Detection of selective sweeps

The selective sweeps were detected using two methods including genetic differentiation (F_ST_) [63] and nucleotide diversity (π). We estimated F_ST_ for each comparison of two chicken populations using VCFtools (0.1.16) [64] with a sliding window size 40 kb and a step size 20 kb. We estimated π for each group using VCFtools (0.1.16) [64] with a sliding window size 40 kb and a step size 20 kb, and calculated the absolute value of the difference in nucleotide diversity (∣Δπ∣) in each window for each comparison of two populations. For both F_ST_ and | Δπ∣, we only used the bi-allelic SNPs on autosomes and sex chromosomes for the analysis. To evaluate the statistic significance of the F_ST_ and π values for a comparison, we generate a Null model by shuffling the allele frequency data for 100 times while keeping the SNP positions fixed [51]. We then computed F_ST_ and ∣Δπ∣ for the permuted windows as well as their means (μ(F_STNull_) and μ(|Δπ|_Null_)) and standard deviations (σ(F_STNull_) and σ(|Δπ|_Null_)). We computed the Z value for each F_ST_ and | Δπ∣ values for a comparison by using the following formulas:

$$\textrm{Z}{\textrm{F}}_{\textrm{ST}}\left(\textrm{i}\right)=\Big({\textrm{F}}_{\textrm{ST}}\left(\textrm{i}\right)-\upmu \left({{\textrm{F}}_{\textrm{ST}}}_{\textrm{Null}}\right)\Big)/{\upsigma \left({{\textrm{F}}_{\textrm{ST}}}_{\textrm{Null}}\right)}\textrm{and}$$

$$\textrm{Z}\left|\Delta \uppi \left(\textrm{i}\right)\right|=\Big(\mid \Delta \uppi \left(\textrm{i}\right)\mid -\upmu \left({\left|\Delta \uppi \right|}_{\textrm{Null}}\right)\Big)/\upsigma \left({\left|\Delta \uppi \right|}_{\textrm{Null}}\right).$$

We consider a window with either ZF_ST_(i) > 6 or Z ∣ Δπ∣ > 3.09 (*P*-value < 0.001) to be a putative selective sweep. Since adjacent putative selective sweep windows may overlap with each other, we merged adjacent windows if they overlapped by at least one nucleotide to obtain the discrete selective sweeps (DSSs) for each comparison.

### Selective sweeps analysis

To reveal selective sweeps related to different major traits of the domestic chicken populations, we conducted a total of 19 comparisons as summarized in Table 1.

**Table 1:**
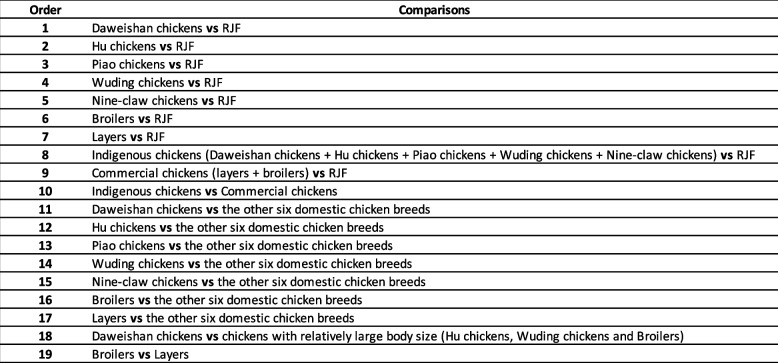
Definitions of the 19 comparisons between different chicken breeds for predicting selective sweeps

## Results

### Indigenous chicken breeds have higher nucleotide diversity

Using the re-sequencing short reads of 25 Daweishan chickens, 10 Hu chickens, 23 Piao chickens, 23 Wuding chickens, four Nine-claw chickens, 60 broilers (two populations), 56 layers (three populations) and 35 RJFs (two populations), we detected 20.4, 15.7, 22.0, 19.5 and 13.0 million single nucleotide variants (SNVs) and small indels (< 50 bp) in Daweishan, Hu, Piao, Wuding and Nine-claw chickens (Table 2), respectively. Taking the union of the SNPs found in the different breeds, we ended up with a total of 30 million non-redundant variants in the indigenous chickens (Table 2). Moreover, consistent with the earlier report [52], we found 16.7, 14.4 and 22.3 million SNVs and small indels in the broilers, layers and RJFs (Table 2), respectively, with a total of 27 million non-redundant variants in them. The five indigenous chickens share 20 million (66.7%) variants with the broilers, layers and RJFs. Thus, there are 10 million (33.3%) variants in the five indigenous chickens that are not seen in the broilers, layers and RJFs. Moreover, the broilers and layers have a total of 18.7 million non-redundant variants (Table 2). There are 17.3, 13.5, 18.7, 16.5, 11.2, 14.1, 12.1 and 19.1 million bi-allelic SNPs in the Daweishan, Hu, Piao, Wuding, Nine-claw chickens, broilers, layers and RJFs populations, respectively (Table 2), with a total of 26 million non-redundant bi-allelic SNPs in them. Our subsequent analyses will be focused on these bi-allelic SNPs (Table 2).

**Table 2:**
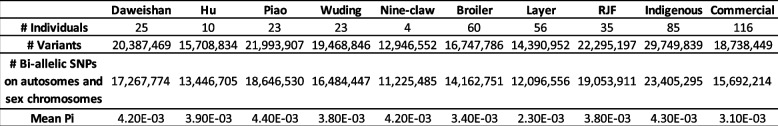
Summary of genetic variants in the chicken groups

We analyzed the mean nucleotide diversity (π) of each chicken breed. Even though both the broilers and the layers were from at least two different sources (Materials and Methods), both had smaller π values than did the five indigenous chickens and RJFs as individual groups or combined groups (Table 2), indicating that commercial chickens have undergone more selection than indigenous chickens, consistent with the breeding histories of commercial and indigenous chickens. The relatively low π values of commercial lines might be due to their close mating and linked selection [52]. Unexpectedly, the mean π value of the indigenous group is higher than that of the RJFs group with two different origins (India and Thailand, Materials and methods, Table 2).

**Fig. 1:**
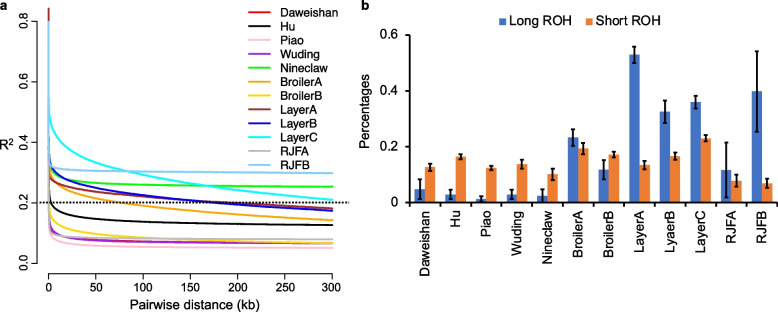
Genetic linkage disequilibrium (LD) and runs of homozygosity (ROHs) in 12 chicken populations. **a.** LD decay in each chicken population as change in average correlation (R^2^) between SNPs among all individuals per population as physical distance increases between SNPs. The dashed horizontal line indicates the R^2^ = 0.2 line to define the LD decay rate (Materials and Methods). **b**. Percentages of ROH on autosomes. Blue bars show fractions of long ROH ≥0.5 Mb, and yellow bars fractions of short ROH < 0.5 Mb. Error bars are SD

To see the degree of inbreeding of the 12 chicken populations (Materials and Methods), we calculated the average LD for each population as change in average correlation (R^2^) between SNPs among all individuals in the population as physical distance increases between SNPs. As shown in Fig. 1a, LD decays much faster in commercial populations than in the indigenous populations, except in nine-claw population where LD decays slower only than in Layer C (crossbred layers), and in Hu population where LD decays faster than in Broiler B (from Indian River International). Specifically, the average distance for the LD to decay until R^2^ = 0.2 (LD decay rate) was 197 kb for Layer A (while white egg layer), 177 kb for Layer B (Brown egg layers), 300 kb for Layer C, 78 kb for Boiler A (from France), and 0.6 kb for Broiler B, while it is 300 kb for nine-claw, and only 2.7 kb for Hu, 0.3 kb for Wuding, 0.2 kb for Daweishan and 0.06 kb for Piao. The slow LD decay rate of nine-claw chickens might be due to their small sample size (*n* = 4) and possible sampling bias. Interestingly, although RJF A (from northern Thailand) has a similarly faster LD decay rate (0.07 kb) to those of Piao and Daweishan populations, RJF B (from India) has very slow LD decay rate > 300 kb (Fig. 1a), indicating their possible high degree of inbreeding. Largely consistent with the LD results, the fraction of runs of homozygosity (ROH) is far greater for the autosomes of commercial chickens than indigenous chickens, particularly, for long ROH (> = 500 kb) (Fig. 1b), indicating a higher degree of recent inbreeding of the commercial chickens than indigenous chickens. Moreover, RJF B has high fraction of long ROH similar to that of commercial chicken populations (Fig. 1b), further indicating that RJF B has a high degree of inbreeding.

### Variants are enriched in non-coding regions while those in coding regions are largely tolerant

Based on the location of the bi-allelic SNPs, we classified them into seven categories, including intergenic (variants in intergenic regions), intronic (variants in introns), up/downstream (variants within a 1 kb region up/downstream of transcription start/end sites), splicing (variants within 2 bp of a splicing junction), UTR 3′/UTR 5′ (variants in 3′/5′ untranslated regions), ncRNA (variants in non-coding RNA genes) and coding (variants in coding sequences). The relative abundances of the bi-allelic SNPs in each chicken group are shown in Table S1. Specifically, of the bi-allelic SNPs in each chicken group, 29.71% ~ 30.46% fall within intergenic regions, 50.44% ~ 51.57% are located in intronic regions, 2.86% ~ 2.97% fall within up/downstream regions, 4.08% ~ 4.24% are located in 3′ UTR/5′ UTR regions, 0.01% fall within splicing regions, 10.09 ~ 10.18% are located in ncRNA regions, and 1.53% ~ 1.80% fall within coding regions (Table S1). Therefore, only a small portion (1.53% ~ 1.80%) of the bi-allelic SNPs fall within coding regions, while the vast majority (98.20% ~ 98.47%) are located in non-coding regions. As non-coding regions comprise 96.92% of the reference chicken genome (GRCg7b assembly), the SNPs are enriched in non-coding regions relative to in coding regions.

Among the bi-allelic SNPs in coding regions of each chicken group, 33.33% ~ 38.33% (Daweishan = 35.71%, Hu = 34.55%, Piao = 36.47%, Wuding = 35.71%, Nine-claw = 33.33%, broiler = 34.97%, layer = 35.22%, RJF = 35.80%, indigenous = 38.33%, commercial = 36.36%) are amino acid-altering (AA-altering, i.e., nonsynonymous and stop-gain/loss) SNPs (Table S1). Among the nonsynonymous SNPs in each chicken group, most (Daweishan = 86.44%, Hu = 87.50%, Piao = 85.24%, Wuding = 86.44%, Nine-claw = 88.00%, broiler = 87.50%, layer = 87.27%, RJF = 85.96%, indigenous = 63.24%, commercial = 69.49%) are tolerant SNPs and only a small proportion (Daweishan = 13.56%, Hu = 12.50%, Piao = 14.76%, Wuding = 13.56%, Nine-claw = 12.00%, broiler = 12.50%, layer = 12.73%, RJF = 14.04%, indigenous = 36.76%, commercial = 30.51%) are intolerant, which might be deleterious variants and thus under purifying selection in the chicken group (Table S1).

### Indigenous chicken breeds have a high portion of rare nonsynonymous SNPs

**Fig. 2:**
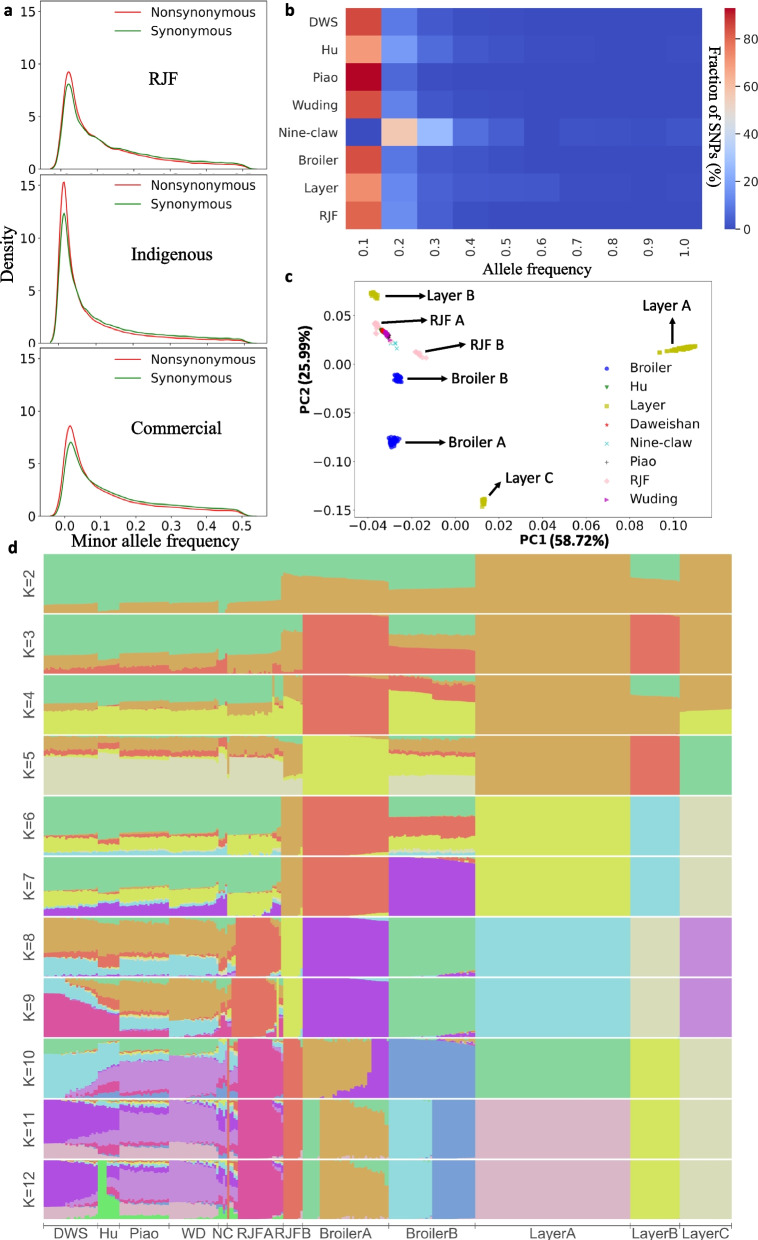
Analysis of frequency spectrums of SNPs. **a.** Distribution of minor allele frequency of synonymous and nonsynonymous SNPs in different chicken groups. **b.** Heatmap of allele frequency of group-specific SNPs. **c.** Principal component analysis of chicken population based on the detected 26 million SNPs. **d.** Genetic structure of the 12 chicken populations estimated using the ADMIXTURE program for K = 2, 3, …, 12

We compared the allele frequencies of the SNPs in coding regions in the groups of RJFs, indigenous and commercial populations. As shown in Fig. 2a, all the three groups have higher portion of rare (< 0.005) nonsynonymous SNPs than rare synonymous SNPs, but the opposite is true for more frequent (> 0.01) ones, indicating that rare nonsynonymous SNPs tend to be deleterious and thus under purifying selection. The same conclusion has been drawn in an earlier study for commercial chickens [52]. Interestingly, the indigenous chickens have the highest rare allele frequency densities for both nonsynonymous and synonymous SNPs, followed by RJFs and the commercial chickens. The earlier study also noted that RJFs had higher rare allele frequency densities than commercial chickens [52]. Among the five different indigenous chicken breeds and two commercial chicken breeds, Piao chickens and the layers have the highest and the lowest rare allele densities (Fig. S1), consistent with their highest and lowest π values, respectively (Table 2).

### Only a small portion of breed-specific SNPs are fixed

We analyzed the group-specific SNPs in each chicken group, and found that RJFs have the highest number of unique SNPs (2.9 million) among the eight chicken groups (Table S2), which is consistent with the finding in the previous study [52], suggesting a loss of ancestral alleles during chicken domestication. Except for Hu chicken and Nine-claw chicken with a small population size (Table 2), layers have the lowest number of unique SNPs (455 k) and broilers have the second lowest number of unique SNPs (520 k) among the eight chicken breeds (Table S2), while Daweishan, Piao and Wuding chickens have 1.1, 1.3 and 0.7 million unique SNPs, respectively, indicating once again that genetic diversity of indigenous chickens is higher than those of the layers and the broilers. From 0.83% (RJFs) to 1.39% (Wuding chicken) of the group-specific SNPs are missense mutations (Table S2). Most of the group-specific SNPs have allele frequencies lower than 0.5, and only a very small portion (0.05% ~ 0.59%) are close to being fixed (allele frequency > 0.9) in all the eight groups of chickens (Table S2). The same is true for the missense SNPs (0.04% ~ 0.48%) (Table S2). More details of the group-specific missense SNPs and affected genes are listed in Tables S3–S10.

We also compared allele frequency spectrums of the group-specific SNPs in the eight chicken groups (Fig. 2b). Group-specific alleles of the layers tend to have higher frequencies than those in other groups (except for Hu chicken and Nine-claw chicken with a small population size), consistent with the finding in the earlier study [52].

### The indigenous chickens are more closely related to one another while commercial chickens are genetically different

To reveal the genetic relationships of the individual chickens, we performed a PCA based on occurring patterns of the 26 million bi-allelic SNPs. As shown in Fig. 2c, the five indigenous populations from Yunnan Province, China, are clustered together with the RJF A population from northern Thailand that is geographically close to Yunnan Province, China, while the RJF B population from India form a separate cluster nearby. On the other hand, Layer B and Broiler B populations form two tight clusters close to the cluster of indigenous chickens and RJF A populations, while Layer A, Layer C and Broiler A populations form three distinct clusters far away from the cluster of indigenous chickens and RJF A populations.

We also analyzed genetic structure of the 12 chicken populations based on the 26 million bi-allelic SNPs. As shown in Fig. 2d, when K = 7, 8 or 9, Broiler A, Broiler B, Layer A, Layer B, Layer C, RJF A and RJF B populations form distinct clusters with little admixture among them, indicating that each of these populations have distinct genetic variations. However, when K = 11 and 12, both Broiler A and Broiler B populations are further clustered in two distinct subclusters, with small admixture of a Broiler A subcluster in the other Broiler A subcluster (Fig. 2d), indicating that each of the two broiler populations are actually made up of two distinct subpopulations. In contrast, none of the four indigenous chicken populations form a distinct cluster for all K > 2 evaluated (Fig. 2d). Instead, all the five the indigenous populations have similar genetic admixture and share small portions of genetic variations with RJF A (Fig. 2d). Both PCA and admixture analyses indicate that the five indigenous breeds are closely related to one another, and they are also more closely related to RJF A from northern Thailand than to RJF B from India, while commercial chickens are genetically different even for those with similar productivities.

### A rigorous null model facilitates sensitive detection of selective sweeps

**Fig. 3:**
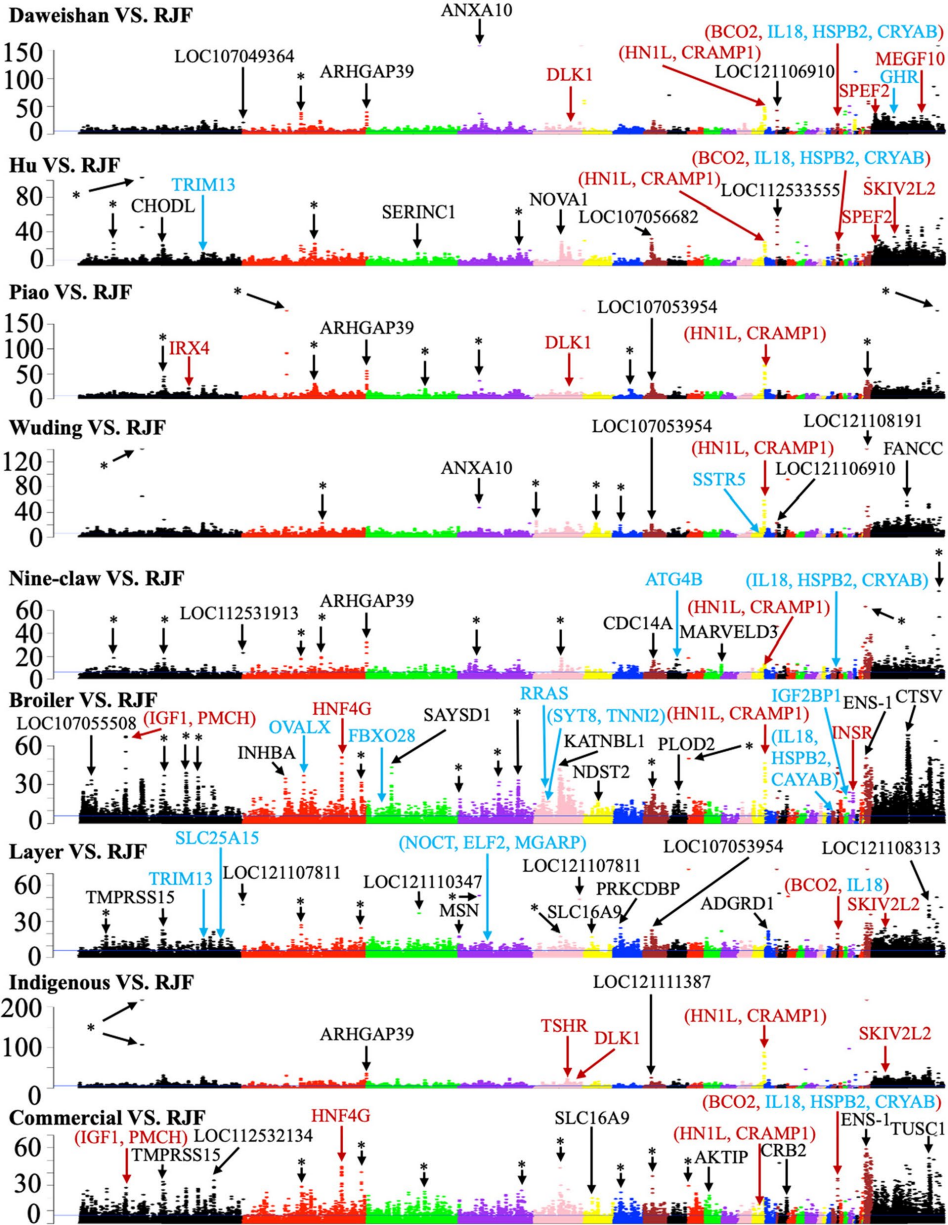
Manhattan plots of ZF_ST_ values of each window on each chromosome for the indicated comparisons. The blue horizontal line indicates the ZF_ST_ cutoff = 6. Examples of genes in significant selective sweep windows are shown in different color. Genes that have been previously reported in selective sweep windows are shown in red, genes in our predicted selective sweep windows potentially related to the specific traits of each chicken breed are shown in blue, and genes in novel selective sweep windows with extremely high ZF_ST_ values are shown in black. Asterisk represents selective sweep windows lacking annotated genes

**Fig. 4:**
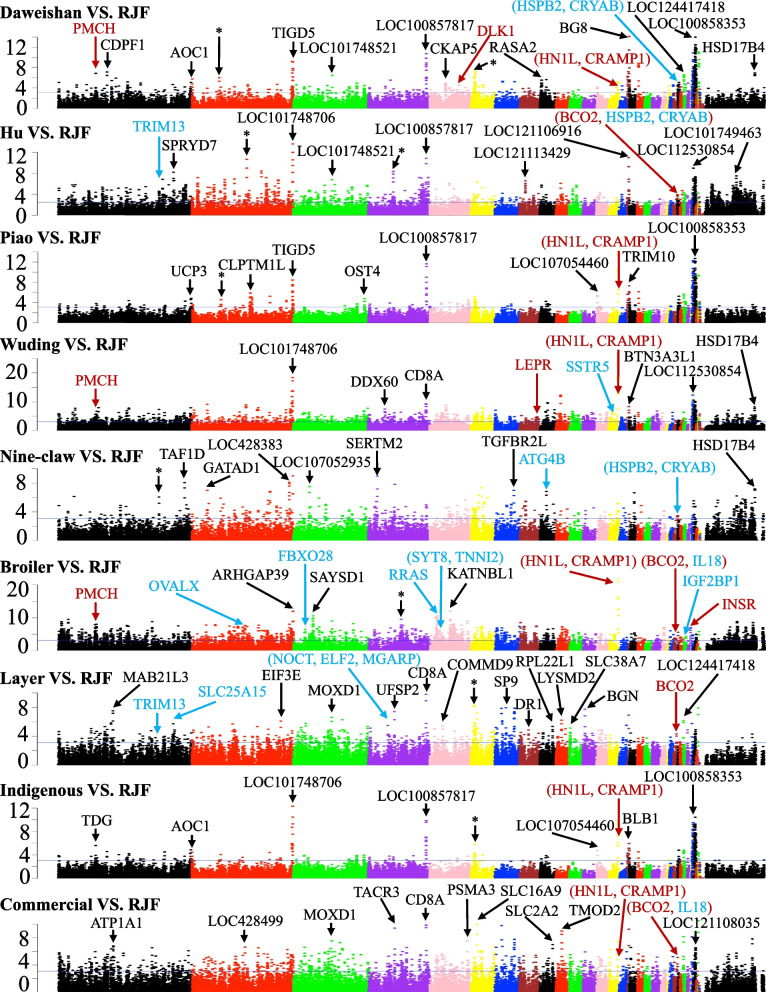
Manhattan plots of Z ∣ Δπ∣ values of each window on each chromosome for the indicated comparisons. The blue horizontal line indicates the Z ∣ Δπ∣ cutoff = 3.09. Examples of genes in significant selective sweep windows are shown in different color. Genes that have been previously reported in selective sweep windows are shown in red, genes in our predicted selective sweep windows potentially related to the specific traits of each chicken breed are shown in blue, and genes in novel selective sweep windows with extremely high Z ∣ Δπ∣ values are shown in black. Asterisk represents selective sweep windows lacking annotated genes

To detect selection signatures of each chicken breed, we identified selective sweeps [65] along each chromosome based on the frequencies of the bi-allelic SNPs using both the F_ST_ and π statistics. We found (see below) that ZF_ST_ is more sensitive than Z ∣ Δπ∣ to identify selective sweeps, thus a higher cutoff of ZF_ST_ (6 vs 3) is used here. Since adjacent windows can overlap with each other, we merged the overlapping selective sweep windows to form a discrete selective sweep (DSS) (Materials and methods). To find selection signatures of the chicken groups, we conducted a total of 19 different comparisons (Tables 3 and S11). The selective sweep windows identified by either of the two methods are distributed along all the chromosomes with varying densities (Figs. 3, 4, S2 and S3). We generally detected more DSSs using ZF_ST_ (806 ~ 2125 DSSs) than using Z|Δπ| (110 ~ 818 DSSs) for all the 19 comparisons (Tables 3 and S11) even using a higher ZF_ST_ cutoff. However, less than 60% (9.16% ~ 58.23%) of the DSSs identified by Z|Δπ| overlap with those identified by ZF_ST_ (Tables S12 ~ S30) in the 19 comparisons, indicating that the results of the two methods are largely complementary with each other. We thus take their union as the final prediction of DSSs (Tables S12–S30). We finally identified 1073 ~ 2745 DSSs consisting of 1998 ~ 7284 windows containing 528 ~ 2147 genes in each of the 19 comparisons (Tables 3 and S11). Therefore, we find much more selective sweeps than the previous study using a mixture model (~ 60 selective sweep windows of 40 kb size) [52]. The DSSs in the 19 comparisons have a varying length ranging from 40 kbp to 2240 kbp with a median length of 60 kbp, and 91.71% of them are shorter than 140 kbp (Fig. S4a). The total length of the DSSs in each comparison consist of 5.85% (Nine Claw vs RJF) ~ 18.88% (Broiler vs Layer) of the reference genome (GRCg7b assembly) (Fig. S4b). In general, comparisons with broilers alone as one of the two compared groups tend to have a high portion of genome under selection (Fig. S4b), suggesting that broilers have gone through most extensive selection.

**Table 3:**
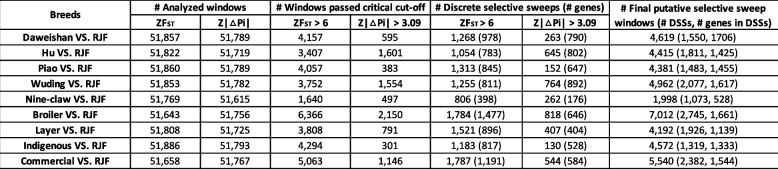
Summary of putative selective sweeps and DSSs found in each chicken group in comparison with the RJFs

### The 19 comparisons reveal selection signatures of the chicken groups

Firstly, to find the genetic differences between artificial selection and natural selection, we compared each chicken breed (including indigenous chicken group and commercial chicken group) with the RJFs. As summarized in Table 3 and Tables S12 ~ S20, we identified varying numbers of selective sweeps (1998 ~ 7012) and DSSs (1073 ~ 2745) involving 528 ~ 1706 genes for the eight comparisons, suggesting that these chicken breeds might have gone through different levels of artificial selection. For example, the Broiler vs RJF comparison yields the highest numbers of selective sweeps (7012) and DSSs (2745), suggesting again that broilers might have gone through the most intensive artificial selection. Among the five indigenous breeds, Wuding chickens might have gone through the most intensive artificial selection with the highest numbers of selective sweeps (4962) and DSSs (2077). In addition, we identified more selective sweeps (5540) and DSSs (2382) for the Commercial vs RJF comparison than those (4572 selective sweeps and 1319 DSSs) in Indigenous vs RJF comparison (Table 3), suggesting that the commercial chickens have gone through more intensive artificial selections than indigenous chickens as generally understood. Interestingly, hundreds of affected genes in putative selective sweeps in each indigenous breed were also found in putative selective sweeps in broilers and layers (Fig. S5).

Secondly, to reveal the genetic differences between traditional selection and industrial selection, we compared the indigenous chicken group with the commercial chicken group and identified a large number of selective sweeps (6735) and DSSs (2532) involving 2147 genes (Tables S11 and S21). This result suggests that indigenous chickens and the commercial chickens have gone through quite different artificial selection routes as commonly understood.

Thirdly, to reveal unique selective sweeps of each chicken breed, we compared each domestic chicken breed with the rest domestic chicken breeds and found that broilers and layers have much higher number of unique selective sweeps (7239 and 6401, respectively) and DSSs (2514 and 2479, respectively) than the indigenous breeds (2801 ~ 4555 and 1363 ~ 1890, respectively) (Tables S11 and S22 ~ 28).

Fourthly, to reveal possible selective sweeps underlying the miniature body size of Daweishan chicken, we compared Daweishan chicken with the group of Hu chicken, Wuding chicken and Broiler (HWB), with a relatively large body size, and identified 6359 selective sweeps and 2263 DSSs including 1911 genes (Tables S11 and S29).

Finally, to find the selection difference between broilers and layers, we compared the two groups and identified 7284 selective sweeps and 2630 DSSs including 1802 genes (Tables S11 and S30). For the similar comparison in a previous study [52], only 41 selective sweeps (40 kb) were identified using a mixture model for normalizing F_ST_ and Δπ. Therefore, we identified substantially more selective sweeps by employing a more rigorous Null model.

### Amino-acid altering SNPs are enriched in the selective sweeps

To identify selective sweeps that might be responsible for the formations of the indigenous chickens, the broiders and the layers, we took the union of DSSs found in comparisons with a breed alone as one of the compared group, e.g., for Daweishan chicken, we took the union of DSSs in comparisons Daweishan vs RJF, Daweishan vs other-breeds and Daweishan vs HWB; and for Hu chicken, we took the union of DSSs in comparisons Hu vs RJF and Hu vs other-breeds; and so on. We identified from 1.2 million (Nine-claw chicken) to 4.1 million (broilers) SNPs in the union of DSSs in each domestic chicken breed (Table S31). Among these SNPs, only 1.3% (Nine-claw chicken) ~ 2.0% (Hu chicken) are located in coding regions, while the remaining vast majority (97.98% ~ 98.72%) fall in non-coding regions (Table S31). As non-coding regions comprise 96.92% of the reference chicken genome (GRCg7b assembly), as in the case of all the bi-allelic SNPs (Table S1), the SNPs in the DSSs are also enriched in non-coding regions relative to in coding regions. Among the SNPs in coding regions, 36.30% ~ 45.23% are amino-acid altering, which are higher than the corresponding values of all the bi-allelic SNPs (33.33% ~ 38.33%) (Table S1), suggesting that amino-acid altering SNPs are enriched in the selective sweeps relative to all the bi-allelic SNPs (*p* = 0.005, K-S test).

### Our predicted selective sweeps are supported by experimental data

To evaluate our detected selective sweeps, we first compared them (Tables S12 ~ S30) with the 15,439 QTLs in the chicken QTL database [66]. We find that 90.5% ~ 98.3% putative DSSs in each of our comparisons overlap one or more QTLs in the chicken QTLdb (Tables 4 and S32). On the other hand, we find that 23.9% ~ 41.6% QTLs in chicken QTLdb overlap one or more our predicted DSSs in each of our comparisons (Tables 4 and S32), and 11,449 (74.2%) QTLs in the chicken QTLdb overlap one or more of our predicted DSSs in different comparisons.

**Table 4:**
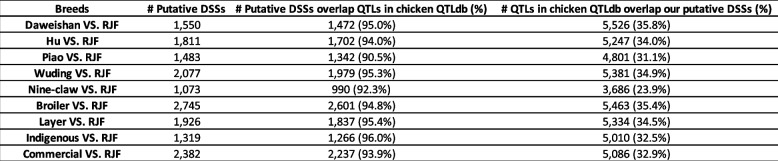
Summary of putative DSSs overlapped with chicken QTLs

As an additional validation of our detected selective sweeps, we next compared the genes in our predicted DSSs with those that have been reported to be under selection during chicken domestication process, and we describe a few examples here. 1) It has been shown that the BCO2 locus is involved in the yellow skin trait in domestic chickens [67], and we confirmed this results in several of our comparisons, including Daweishan vs RJF (ZF_ST_ = 11.27), Daweishan vs HWB (ZF_ST_ = 14.79), Hu vs RJF (ZF_ST_ = 18.26, Z ∣ Δπ∣ = 4.86), Hu vs other-breeds (ZF_ST_ = 6.27), Piao vs other-breeds (ZF_ST_ = 29.36), Wuding vs other-breeds (ZF_ST_ = 22.18), Broiler vs RJF (Z ∣ Δπ∣ = 5.08), Broiler vs Layer (ZF_ST_ = 9.90), Layer vs RJF (ZF_ST_ = 15.66, Z ∣ Δπ∣ = 3.17), Layer vs other-breeds (ZF_ST_ = 8.70), Indigenous vs Commercial (ZF_ST_ = 26.75, Z ∣ Δπ∣ = 3.67) and Commercial vs RJF (ZF_ST_ = 27.16, Z ∣ Δπ∣ = 4.44) (Figs. 3, 4, S2 and S3); 2) The *TSHR* locus is known to be involved in regulation of reproduction and metabolic functions in commercial chickens [53], and we found that the locus was in selective sweeps in comparisons Hu vs other-breeds (ZF_ST_ = 11.75) and Indigenous vs RJF (ZF_ST_ = 13.43) (Figs. 3 and S2); 3) It has been reported that the *HNF4G* and *IGF1* loci are involved in growth regulation in chicken [53, 68], and we found that the two loci were in selective sweeps in comparisons Broiler vs RJF (for HNF4G, ZF_ST_ = 24.27; for IGF1, ZF_ST_ = 32.54), Commercial vs RJF (for *HNF4G*, ZF_ST_ = 26.25; for *IGF1*, ZF_ST_ = 15.51) and Indigenous vs Commercial (for *HNF4G*, ZF_ST_ = 24.51; for *IGF1*, ZF_ST_ = 17.86) (Figs. 3 and S2); 4) It has been reported that the *PMCH* locus is related to regulation of appetite and metabolic functions [53, 69], and we found that the locus was in the selective sweeps in several of our comparisons including Daweishan vs RJF (Z ∣ Δπ∣ = 3.25), Daweishan vs HWB (Z ∣ Δπ∣ = 6.04), Wuding vs RJF (Z ∣ Δπ∣ = 4.82), Wuding vs other-breeds (Z ∣ Δπ∣ = 4.07), Broiler vs RJF (ZF_ST_ = 32.54, Z ∣ Δπ∣ = 6.05), Broiler vs other-breeds (ZF_ST_ = 19.59, Z ∣ Δπ∣ = 6.34), Broiler vs Layer (ZF_ST_ = 27.21), Commercial vs RJF (ZF_ST_ = 15.51) and Indigenous vs Commercial (F_ST_ = 17.86) (Figs. 3, 4, S2 and S3); 5) It has been shown that the INSR locus is related to the growth of chicken by encoding a critical component in insulin signaling [53], and we found that the locus was in the selective sweeps in comparisons Daweishan vs HWB (ZF_ST_ = 7.37, Z ∣ Δπ∣ = 4.46), Broiler vs RJF (ZF_ST_ = 15.30, Z ∣ Δπ∣ = 5.23), Broiler vs other-breeds (ZF_ST_ = 11.61, Z ∣ Δπ∣ = 4.86) and Broiler vs Layer (ZF_ST_ = 9.78) (Figs. 3, 4, S2 and S3); 6) It has been shown that the *NELL*1 locus is related to the skeletal integrity in broiler [68], and we found that the locus was in the selective sweeps of the Broiler vs other-breeds comparison (ZF_ST_ = 22.06) (Fig. S2); 7) It has been reported that the IRX4 locus is related to the rumpless trait of Piao chicken [45], and we found that the locus was in the selective sweeps in comparisons Piao vs RJF (ZF_ST_ = 13.79) and Piao vs other-breeds (ZF_ST_ = 12.12) (Figs. 3 and S2). The other selective sweep loci found in the previous studies are also confirmed by our results, such as *ALX*1, *KITLG, EGFR, DLK*1, *JPT*2 (annotated as *HN1L* in GRCg7b), *CRAMP*1 and *GLI*3 loci, which are related to the general domestication process of chicken [52], the *SKIV2L*2 locus that is related to pigmentation [52], and the *LEPR*, *MEGF*10 and *SPEF*2 loci, which are related to production-oriented selection [52] (Figs. 3, 4, S2 and S3).

### Novel selective sweeps are found in the chicken breeds

In addition to confirming many previously identified selective sweeps containing genes related to chicken domestication as described above, we also find numerous novel selective sweeps containing genes (Tables S12 ~ S30) or in gene deserts. We now highlight a few of them with extremely high ZF_ST_ and/or Z ∣ Δπ∣ values in each comparison (Figs. 3, 4, S2 and S3). 1) Gene ARHGAP39 on chromosome 2 is in the selective sweeps with extremely high ZF_ST_ and/or Z ∣ Δπ∣ values in comparisons Daweishan vs RJF, Piao vs RJF, Nine-claw vs RJF, Indigenous vs RJF, Nine-claw vs other-breeds and Broiler vs RJF. ARHGAP39 plays important roles in cell cytoskeletal organization, growth, differentiation, neuronal development and synaptic functions [70]; 2) Gene TIGD5 on chromosome 2 is in the selective sweeps with extremely high Z ∣ Δπ∣ values in comparisons Daweishan vs RJF, Piao vs RJF, Daweishan vs other-breeds, Hu vs other-breeds, Piao vs other-breeds, Wuding vs other-breeds, Daweishan vs HWB and Indigenous vs Commercial. *TIGD*5 encodes the tigger transposable element-derived protein 5 and is important for nucleic acid binding [71]; 3) Gene *KCNK*16 on chromosome 3 is in the selective sweeps with extremely high ZF_ST_ values in comparisons Layer vs other-breeds and Broiler vs Layer. *KCNK*16 encodes a rapidly activating and non-inactivating outward rectifier K^+^ channel [72]; 4) Gene *CD8A* on chromosome 4 is in the selective sweeps with extremely high Z ∣ Δπ∣ values in comparisons Wuding vs RJF, Layer vs RJF, Commercial vs RJF, Daweishan vs other-breeds, Piao vs other-breeds, Wuding vs other-breeds, Nine-claw vs other-breeds, Broiler vs other-breeds, Layer vs other-breeds, Indigenous vs Commercial and Daweishan vs HWB. CD8A encodes the T-cell surface glycoprotein CD8 alpha chain precursor and plays essential roles in immune response [73]; 5) Gene *COL6A*2 on chromosome 7 is in the selective sweeps with extremely high ZF_ST_ values in comparisons Daweishan vs other-breeds, Daweishan vs HWB and Piao vs other-breeds. The gene encodes the collagen alpha-2(VI) chain precursor which act as a cell-binding protein [74]. Besides the genes mentioned above, we also indicate in Figs. 3, 4, S2 and S3 many other genes located in novel selective sweeps with extremely high ZF_ST_ and/or Z ∣ Δπ∣ values in multiple comparisons such as: *ANXA*10 on chromosome 3, gene *LOC107053954* on chromosome 8, gene *SLC16A*9 on chromosome 6, gene *ENS*-1 on chromosome W and gene *HSD17B*4 on chromosome Z. It is interesting to experimentally investigate the roles of these genes in the domestication and breeding of each chicken breed.

In Figs. 3, 4, S2 and S3, we also label a few examples of selective sweeps in gene desserts, with extremely high ZF_ST_ and/or Z ∣ Δπ∣ values in multiple comparisons. It is highly likely that these selective sweeps might harbor non-coding functional sequences such as cis-regulatory modules of distal genes.

### Selective sweeps related to each chicken breed

In addition to finding numerous novel selective sweeps containing genes in each comparison (Tables S12 ~ S30), we also identify numerous unique selective sweeps/DSSs that are only seen in comparisons with a breed alone as one of the two compared groups or selective sweeps/DSSs containing genes with interesting functions. These selective sweeps/DSSs might contain genes (Tables S33 ~ S39) whose functions are related to the specific traits of the chicken breed. Specifically, for Daweishan chicken, we identified 44 putative genes in the selective sweeps that might be related to its unique traits including the small body size (Table S33). For example, the *GHR* (growth hormone receptor) gene is located in a selective sweep window on chromosome Z, which overlaps body weight QTLs and shank length QTLs. The gene is in the selective sweep windows identified in comparisons with Daweishan chicken alone as one of the two compared groups (Daweishan vs RJF, ZF_ST_ = 17.71; Daweishan vs other-breeds, ZF_ST_ = 17.69; Daweishan vs HWB, ZF_ST_ = 19.51) (Figs. 3 and S2). It has been reported that loss-of-function mutations in GHR was related to sex-linked dwarfism in chicken [75]. We analyzed the SNPs in the GHR gene body for each chicken breeds using the GRCg7b assembly as the template and found 79 unique SNPs in the gene of Daweishan chicken, which were not present in the other chicken breeds (Hu, Piao, Wuding, Nine-claw, Broiler, Layer and RJF). Among these 79 unique SNPs, 68 are in intronic regions, 10 are in UTRs and one is nonsynonymous SNP that leads to a CGG to TGG (R to W) codon mutation, which is predicted to be tolerant by VEP [62]. The substitution allele has a frequency of 0.76, thus it is only nearly fixed. As no fixed potential amino acid-altering mutation could be found in the GHR coding regions, we hypothesize that *GHR* gene might be related to the small body size of Daweishan chicken through changes in its regulatory sequences in the window, resulting in changes in its expression.

For Hu chicken, we identified 14 putative genes in selective sweeps (Table S34) that might be related to its unique traits including the very stout legs. Specifically, gene *TRIM*13 in a selective sweep on chromosome 1 (Hu vs RJF, ZF_ST_ = 7.49 and Z ∣ Δπ∣ = 7.19; Hu vs other-breeds, ZF_ST_ = 10.01 and Z ∣ Δπ∣ = 4.58) (Figs. 3, 4, S2 and S3) overlaps shank circumference QTLs, and there are two nonsynonymous SNPs in the gene body which are fixed (allele frequency = 1). Thus, it is interesting to experimentally investigate the role of *TRIM*13 in the development of the very stout legs of Hu chicken.

For Piao chicken, we identified six putative genes in selective sweeps (Table S35) that might be related its unique traits including the rumpless trait. Of these six genes, *IRX*4 in a selective sweep on chromosome 2 was reported to be related to the rumpless trait of Piao chicken in a previous study [45], and we also found that the selective sweeps were only identified by our comparisons Piao vs RJF (ZF_ST_ = 13.79) and Piao vs other-breeds (ZF_ST_ = 12.12) among the 19 comparisons (Figs. 3 and S2, Tables S14 and S24). Thus, it is highly likely that IRX4 is related to the rumpless trait of Piao chicken. At the same time, the previous study also identified genes *IL*18, *HSPB*2, and *CRYAB* to be related to the rumpless trait of Piao chicken. Although we also found these three genes in the selective sweeps of the comparison Piao vs other-breeds (Table S24 and Fig. S2), these three genes were also present in the selective sweeps of comparisons with a breed having normal tails alone as one of the two compared groups, such as Daweishan vs RJF (Table S12, Figs. 3 and S2), Hu vs RJF (Table S13, Figs. 3 and S2), Nine-claw vs RJF (Table S16, Figs. 3 and S2), Broiler vs RJF (Table S17, Figs. 3 and S2), Layer vs RJF (Table S18, Fig. 3) and Daweishan vs HWB (Table S29, Fig. 3). Therefore, these three genes might not be related to the rumpless trait of Piao chicken.

For Wuding chicken, we identified 18 putative genes in selective sweeps (Table S36) that might be related to its unique traits including colorful feathers and thick fat. Specifically, gene SSTR5 in a selective sweep on chromosome 14 (Wuding vs RJF, ZF_ST_ = 7.38 and Z ∣ Δπ∣ = 5.16; Wuding vs other-breeds, Z ∣ Δπ∣ = 3.33) (Figs. 3, 4 and S3) overlaps body weight QTLs, however, there are no nonsynonymous SNPs in its gene body. Gene *LOC101748311* in a selective sweep on chromosome 1 (Wuding vs other-breeds comparison, ZF_ST_ = 9.70 and Z ∣ Δπ∣ = 3.59) (Figs. S2 and S3) overlaps the feather density QTLs and comb length QTLs and there are two nonsynonymous SNPs in its gene body, but their allele frequencies are very low (< 0.2). It is likely that both genes might be related to Wuding chicken’s traits by changes in regulatory regions, which warrants further experimental studies.

For Nine-claw chicken, we identified seven putative genes in selective sweeps (Table S37) that might be related to its unique traits. Specifically, gene *ATG4B* on chromosome 9 (Nine-claw vs RJF, ZF_ST_ = 7.00 and Z ∣ Δπ∣ = 3.60; Nine-claw vs other-breeds, ZF_ST_ = 7.56) (Figs. 3, 4 and S2) overlaps egg production rate QTLs, but there are no nonsynonymous SNPs in its gene body.

For Broilers, we identified 151 putative genes in selective sweeps (Table S38) that might be related to its unique traits including the fast growth rate. Of these genes, *GHRHR* on chromosome 2 (Growth hormone-releasing hormone receptor) (Broiler vs other-breeds, ZF_ST_ = 10.36; Broiler vs Layer, ZF_ST_ = 7.09) (Fig. S2) is well-known for its role in determining growth rate and body size via regulating the growth hormone (GH) level in blood [76], however, there are no nonsynonymous SNPs in its gene body. IGF2BP1 on chromosome 27 (insulin-like growth factor 2 mRNA-binding protein 1) (Broiler vs RJF, ZF_ST_ = 7.69 and Z ∣ Δπ∣ = 3.98) (Figs. 3 and 4) may affect growth rate via regulating insulin-like growth factor 2 level [77], but there are no nonsynonymous SNPs in its gene body. The IGF2BP1 locus also overlaps the claw percentage QTLs, shank length QTLs, claw weight QTLs, drumstick and thigh weight QTLs, breastbone crest length QTLs, body weight QTLs, body slope length QTLs and femur bending strength QTLs. The result is consistent with a recent report that mutations in the promoter region of the IGF2BP1 gene can affect chicken body size [18]. In addition, the following genes are also interesting as they overlap white striping QTLs, abdominal fat percentage QTLs, wooden breast QTLs and body weight QTLs, and thus might be related to the large body and fast growth rate of broilers, including OVALX on chromosome 2 (Broiler vs RJF, ZF_ST_ = 9.41 and Z ∣ Δπ∣ = 5.78; Broiler vs other-breeds, ZF_ST_ = 8.45 and Z ∣ Δπ∣ = 4.18) (Figs. 3, 4, S2 and S3), RRAS on chromosome 5 (Broiler vs RJF, ZF_ST_ = 14.87 and Z ∣ Δπ∣ = 7.53; Broiler vs other-breeds, ZF_ST_ = 11.73 and Z ∣ Δπ∣ = 6.00) (Figs. 3, 4, S2 and S3), SYT8 on chromosome 5 (Broiler vs RJF, ZF_ST_ = 6.90 and Z ∣ Δπ∣ = 5.87; Broiler vs other-breeds, ZF_ST_ = 6.89 and Z ∣ Δπ∣ = 3.85) (Figs. 3, 4, S2 and S3), *TNNI*2 on chromosome 5 (Broiler vs RJF, ZF_ST_ = 6.90 and Z ∣ Δπ∣ = 5.87; Broiler vs other-breeds, ZF_ST_ = 6.89 and Z ∣ Δπ∣ = 3.85) (Figs. 3, 4, S2 and S3) and FBXO28 on chromosome 3 (Broiler vs RJF, ZF_ST_ = 10.25 and Z ∣ Δπ∣ = 5.30; Broiler vs other-breeds, ZF_ST_ = 8.38 and Z ∣ Δπ∣ = 4.90) (Figs. 3, 4, S2 and S3). All these genes either has no nonsynonymous SNPs or the allele frequencies of their nonsynonymous SNPs are very low. Thus, it is highly likely that they might be related to the broilers’ traits through changes in their regulatory regions.

For layers, we identified 36 genes in selective windows (Table S39) that might be related to its unique traits including larger number of egg-production. Specifically, gene *NOCT* (Nocturnin), ELF2 (ETS-related transcription factor Elf-2) and *MGARP* (mitochondria localized glutamic acid rich protein) are all located in the same selective sweep on chromosome 4 (Layer vs RJF, ZF_ST_ = 9.84 and Z ∣ Δπ∣ = 4.61; Layer vs other-breeds, ZF_ST_ = 10.80 and Z ∣ Δπ∣ = 5.24; Broiler vs Layer, ZF_ST_ = 9.23 and Z ∣ Δπ∣ = 5.20) (Figs. 3, 4, S2 and S3). *NOCT* is expressed in retina and many other tissues, and its expression shows circadian rhythm [78, 79]. *NOCT* is known to be involved in adipogenesis, osteogenesis, and obesity in mice [80]. It has been shown that *MGARP* is involved in the synthesis of estrogen in ovary, and its expression is under the control of the hypothalamic-pituitary-gonadal (HPG) axis [81]. It has been reported that ELF2 plays a role in cell proliferation [82]. Moreover, gene *SLC25A*15 on chromosome 1 (mitochondrial ornithine transporter 1) (Layer vs RJF, ZF_ST_ = 7.23 and Z ∣ Δπ∣ = 3.96; Layer vs other-breeds, ZF_ST_ = 9.68 and Z ∣ Δπ∣ = 3.82; Broiler vs Layer, ZF_ST_ = 10.52 and Z ∣ Δπ∣ = 3.21) (Figs. 3, 4, S2 and S3) overlaps the oviduct length QTLs, thus might be related to the egg-production rate of layers. Thus, these four genes might be related to the layers’ unique traits. However, the four genes either have no nonsynonymous SNPs or the allele frequencies of their nonsynonymous SNPs are very low. Thus, it is highly likely that they might be related to the high egg-production of layer through changes in their regulatory regions.

### Validation of eight genes in DSSs that might be related to body size and egg production rate using RT-qPCR

To test our hypothesis that many genes in DSSs might be related to specific traits through changes in their regulatory sequences in the DSSs, resulting in changes in their expression levels, we used RT-qPCR to quantify the expression levels of eight genes in relevant tissues in chicken breeds with contrast traits.

**Fig. 5:**
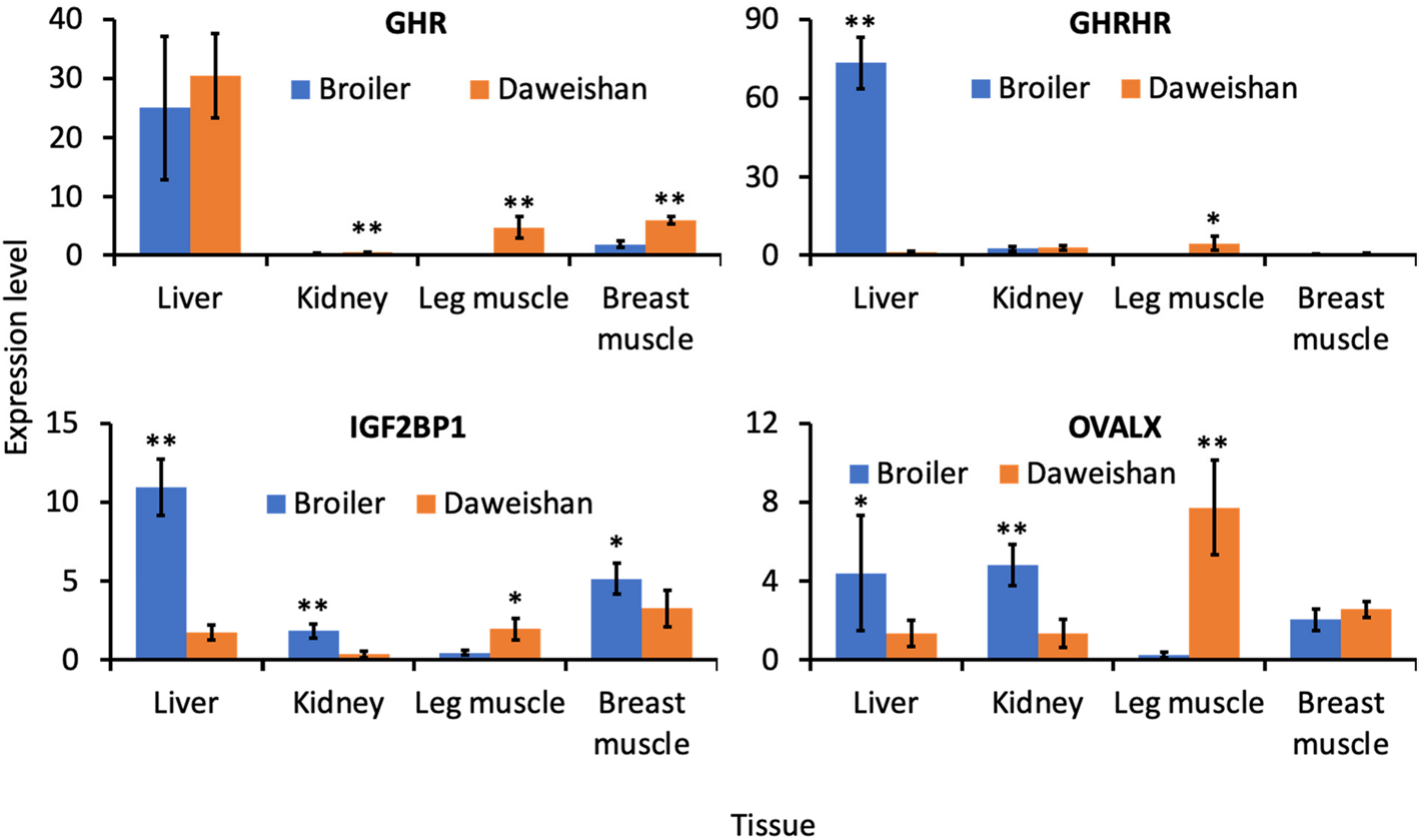
Comparison of expression levels of *GHR, GHRHR, IGF2BP1* and OVALX in different tissues of broilers and Daweishan chickens. *p < 0.05, **p < 0.01, two-tailed t-test

Specifically, as we inferred that *GHR, GHRHR, IGF2BP1* and *OVALX* might be related to body size, we measured their expression levels in the liver, kidney, leg muscle and breast muscle of Daweishan chickens (small body size) and broilers (large body size). As shown in Fig. 5, consistent with our previous results [76], Daweishan chickens had significantly (*p* < 0.01) higher *GHR* expression levels in the kidney, leg muscle and breast muscle tissues than did the broilers. However, it remains to be elucidated how the higher *GHR* expression level is related to the small body size of Daweishan chickens. On the other hand, *GHRH*R had significantly higher (p < 0.01) expression levels in the liver, but significantly lower (*p* < 0.05) expression levels in the leg muscle of broilers than in the same tissues of Daweishan chickens (Fig. 5). Moreover, *IGF2BP1* had significantly higher (p < 0.05 or p < 0.01) expression levels in the liver, kidney and breast muscle, but significantly low (p < 0.05) expression levels in the leg muscle of broilers than in the respective same tissues of Daweishan chickens (Fig. 5). Furthermore, *OVALX* had significantly higher (p < 0.05 or p < 0.01) expression levels in the liver and kidney, but significantly lower (p < 0.01) expression levels in the leg muscle of broilers than in the same tissues of Daweishan chickens (Fig. 5). The differential expression levels of *GHRHR, IGF2BP1* and *OVALX* in the liver, kidney and muscles might be related to the different metabolic and growth rates of the two breeds.

**Fig. 6:**
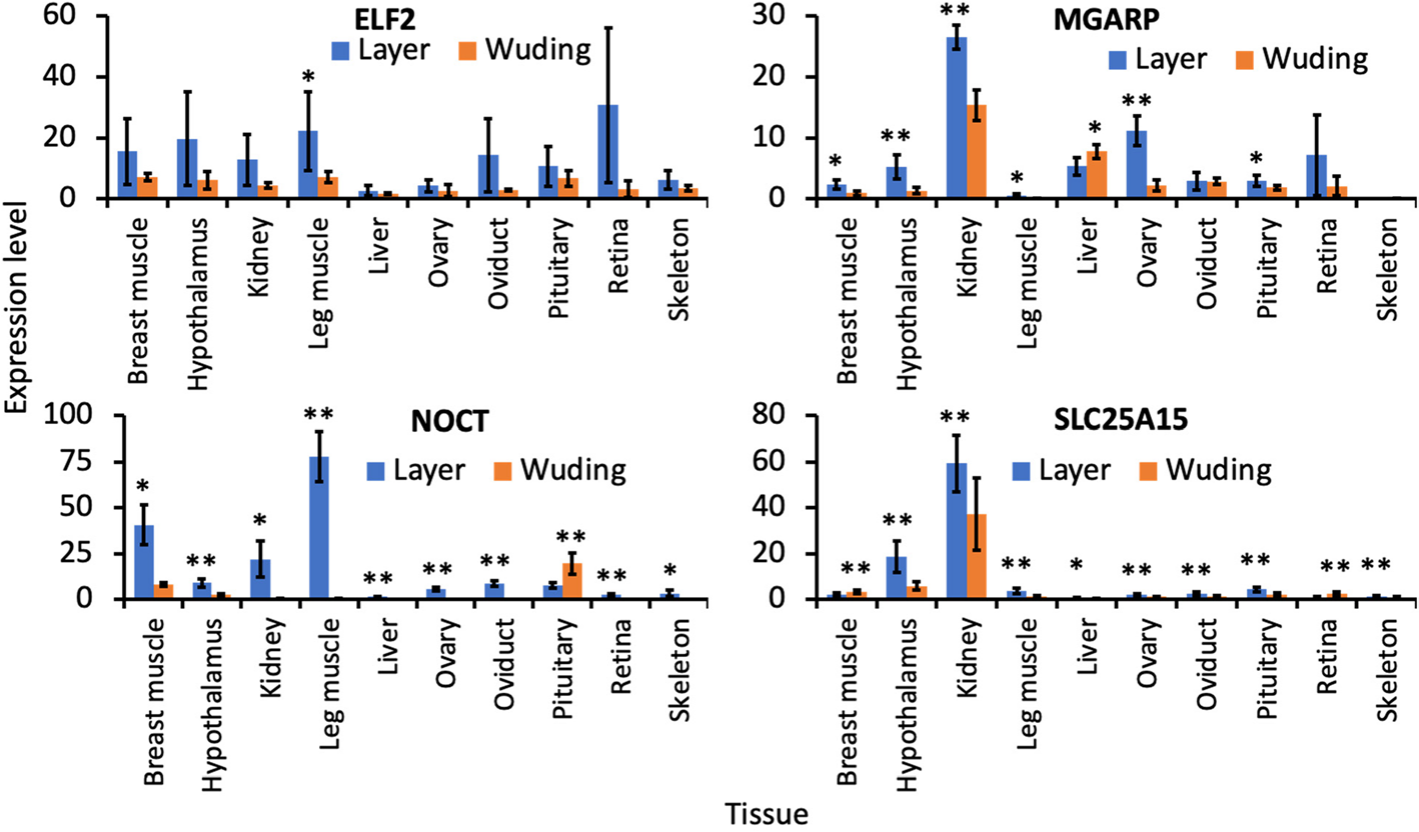
Comparison of expression levels of *ELF2, MGARP, NOCT* and *SLC25A*5 in different tissues of Layers and Wuding chickens. *p < 0.05, **p < 0.01, two-tailed t-test

**Fig. 7:**
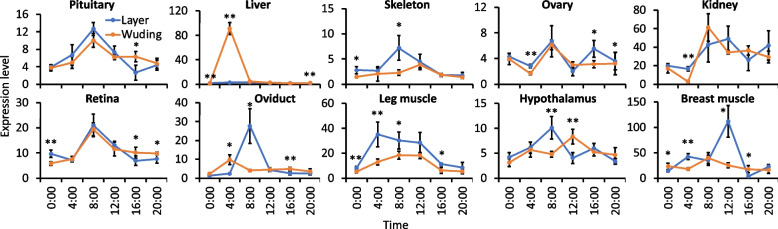
Comparison of expression levels of *NOCT* at different time of a day in different tissues of layers and Wuding chickens. *p < 0.05, **p < 0.01, two-tailed t-test

In addition, as we predicted that *ELF2, MGARP, NOCT* and *SLC25A*15 might be related to the egg production rate, we measured their expression levels in 10 tissues of layers (high-egg production rate) and Wuding chickens (low-egg production rate). As shown in Fig. 6, although *ELF*2 had significantly higher (p < 0.05) expression level only in the leg muscle of layers than in the same tissue of Wuding chicken, *MGARP, NOCT* and *SLC25A15* had significantly higher (p < 0.01 or p < 0.05) expression levels in almost all the 10 tissues of layers than in the same tissues of Wuding chickens, except for *MGARP* in the liver, for *NOCT* in pituitary and for *SLC25A*15 in breast muscle and retina. Moreover, as the expression of *NOCT* shows circadian rhythm [78, 79], we measured its expression level in 10 tissues of layers and Wuding chickens in 24 hours with 4 hours interval. As shown in Fig. 7, in almost all the 10 tissues, the expression level of *NOCT* was lower after 16:00, and it started to increase at 4:00, peaked at 8:00, and then decreased. In most tissues, layers had significantly higher (p < 0.05 or p < 0.01) expression levels at certain time points than did Wuding chickens. The higher expression level of these four genes in relevant tissues in layers might be related to their high egg-production trait.

## Discussion

Next generation sequencing technology makes it possible to re-sequence a large number of individuals for a species for genome-wide studies. In 2021, NCBI released more complete domestic chicken (*Gallus gallus*) genome assemblies (GRCg7b and GRCg7w), providing good reference genomes for this economically, medically and evolutionally important bird. Using the GRCg7b assembly as the template, we called the variants in populations of five indigenous chicken breeds, broilers, layers and RJFs. By comparing the putative selective sweeps of Daweishan, Hu, Piao, Wuding, Nine-claw chicken, broilers and layers with respect to others and RJFs (19 comparisons, Tables 3 and S11), we identified putative selective sweeps and genes that might be related to the specific traits of each chicken breed or groups (Tables S33 ~ S39). Remarkably, the vast majority (90.5% ~ 98.3%) of our identified DSSs in each of our 19 comparisons overlap QTLs in the chicken QTLdb (Tables 4 and S32), while 74.2% QTLs in the chicken QTLdb overlap our identified DSSs in different comparisons, suggesting that our approach of finding selective sweeps is quite sensitive. Moreover, we also confirm many previously identified genes under artificial selection. Taken together, we have achieved very high prediction precision (or positive prediction values) and sensitivity.

More importantly, our analyses also result in many new findings. Firstly, our results indicate that the RJF A population from northern Thailand are genetically different from those (RJF B) from India. Moreover, the RJF A population are genetically more diverse than the RJF B population due probably to the latter’s higher degree of recent inbreeding on the farms since their ancestor’s capture as indicted by their higher proportion of long ROH. Both PCA and genetic structure analyses indicate that the five indigenous breeds are closely related to one another, and also more closely related to RJF A than to RJF B. On the other hand, the three layer populations (Layer A, Layer B and Layer C) with different origins have quite different genetic structures though they all are selected for high egg productivity. The same is true for the two broilers populations (Broiler A and Broiler B) though both are selected for high meat productivity. Furthermore, we find that indigenous chickens have higher density of rare allele frequencies for nonsynonymous SNPs than the commercial chickens. As rare alleles tend to be deleterious, the indigenous chickens are more likely to harbor deleterious mutations than commercial chickens. Intensive industrial selective breeding of commercial chickens might lead to the loss of rare alleles which might be slightly deleterious.

Secondly, we identify a much larger number of selective sweeps/DSSs and genes related to the specific traits of broilers and layers than the previous study [52]. We attribute the difference to the different statistic models used in the two studies. More specifically, we use a more rigorous Null model by generating 100-sets of windows with the allele frequencies randomly permutated [6, 51]. Using the mean and standard deviation of the Null model, we compute ZF_ST_ and Z ∣ Δπ∣ for each window in each comparison. In contrast, the previous study [52] used the mean and standard deviation of the F_ST_ and ∣Δπ∣ values of the windows to compute the ZF_ST_ and Z ∣ Δπ∣, which is not a rigorous Null model. Thus, the previous study might underestimate the number of selective sweeps. Consequently, we identify ~ 2500 putative DSSs containing ~ 1800 genes for the broilers and ~ 2000 putative DSSs containing ~ 1000 genes for the layers (Tables 3 and S11), which included almost all the only 90 and 66 putative selective sweep windows (40 kb) found in broilers and layers, respectively, in the previous study [52]. Notably, we were able to identify more selective sweeps using ZF_ST_ than using Z ∣ Δπ∣, suggesting that the former might be more sensitive than the latter to identify selective sweeps. In the comparisons between each domestic chicken breed and RJFs, we found numerous genes that might be under selection in both indigenous chickens and commercial chickens, indicating that these genes might be beneficial for the chickens to live in both traditional and industrial artificial conditions.

Thirdly, we negate several genes reported to be related to the rumpless trait of Piao chicken by a previous study [45] based on our results from multiple comparisons with or without Piao chicken. More specifically, in addition to *IRX*4, the previous study also claimed that *IL18, HSPB2*, and *CRYAB* [45] might be related to the rumpless trait of Piao chicken. As we also find that gene IRX4 is present in putative selective sweeps only in the Piao vs RJF and Piao vs other-breeds comparisons among our 19 comparisons (Tables S14 and S24), it might be related to the rumpless trait of Piao chicken. However, genes IL18, HSPB2, and CRYAB are present in selective sweeps in not only the comparison related to Piao chicken (Piao vs other-breeds, Table S24), but also in comparisons with chickens having a normal tail alone as a group, such as Daweishan, Wuding, Nine-claw chicken, broilers and layers (Tables S12 and S15 ~ S18). Thus, these three genes might not be related to the rumpless trait of Piao chicken.

Fourthly, our analyses provide many novel selective sweeps containing genes that might be related to artificial selection of unique traits of each chicken breed (Tables S33 ~ S39), and some are quite interesting, thus warranting further experimental investigations. For example, it is interesting to experimentally further investigate variations in the regulatory regions of *NOCT* and *MGARP* for their roles in the high egg-production related traits of layers, such as the lack of brooding behaviors, egg-laying circadian rhythm and high demand for light.

Finally, we find that although SNPs in selective sweeps are more likely to alter amino acids than expected, many genes in selective sweeps often lack fixed amino acid-altering mutations. We found that SNPs in non-coding regions in general, or in selective sweeps in particular, are enriched in all the seven domestic chicken breeds as well as RJF analyzed in this study. Thus, most genes in selective sweeps might affect the traits of chicken breeds by changing their expression levels through changes in their cis-regulatory regions. Consistently, we found that all the eight genes (*GHR, GHRHR, IGF2BP1, OVALX, ELF2, MGARP, NOCT, SLC25A15*) that we examined were significantly differentially expressed in relevant tissues of chicken breeds where these genes were in putative selective sweeps from those of chicken breeds where these genes were not in selective sweeps. Particularly, it was recently found that two different forms of deletions in the upstream region of the *IGF2BP1* gene affected body weight of various chicken breeds [18], and we found that the expression levels of the gene in relevant tissues of broilers differed significantly from those in the same tissues of Daweishan chickens (Fig. 5). However, due to the lack of a more complete map of *cis*-regulatory modules and their constituent transcription factor binding sites in the chicken reference genome, it is difficult to further pin down the sites that affect the expression of these genes and related organism traits. Therefore, it is pressing to map out the *cis*-regulatory elements in the chicken reference genome as has been done for *C. elegans* [83, 84], *D. melanogaster* [83, 85], mice [86, 87] and humans [87, 88].

## Conclusions

We identify 30 million single nucleotide variants and small indels in the five indigenous chicken breeds, 10 million of which are novel. Using a rigorous statistic model, we are able to predict substantially more selective sweeps and affected genes than previously reported in both indigenous and commercial breeds. We not only confirm most of previously identified selective sweeps and affected genes in commercial chickens, but also identify numerous novel candidates that might be related to the unique traits of each breed. Most of the variants in selective sweeps are located in non-coding regions and overlap known chicken QTLs, and they might affect the traits of chicken breeds by changing their expression levels through mutations in their *cis*-regulatory elements. Our results can be beneficial to breeding programs in chicken industry for more efficient and secure production of eggs and meat.

### Supplementary Information

**Supplementary Material 1.**

**Supplementary Material 2.**

**Supplementary Material 3.**

## Data Availability

All the re-sequencing data of the indigenous chickens are available in the SRA database with the accession number of ‘PRJNA893352’ (https://www.ncbi.nlm.nih.gov/sra/?term=PRJNA893352).
