# Supplementary material for: Artificial selection footprints in indigenous and commercial chicken genomes
Source: BMC Genomics. 2024 Apr 30;25:428. doi: 10.1186/s12864-024-10291-5 (PMC11061962; doi:10.1186/s12864-024-10291-5)
Supplement: Supplementary file 1 — Supplementary Material 1. [file 12864_2024_10291_MOESM1_ESM.docx]

**Supplementary Figures**


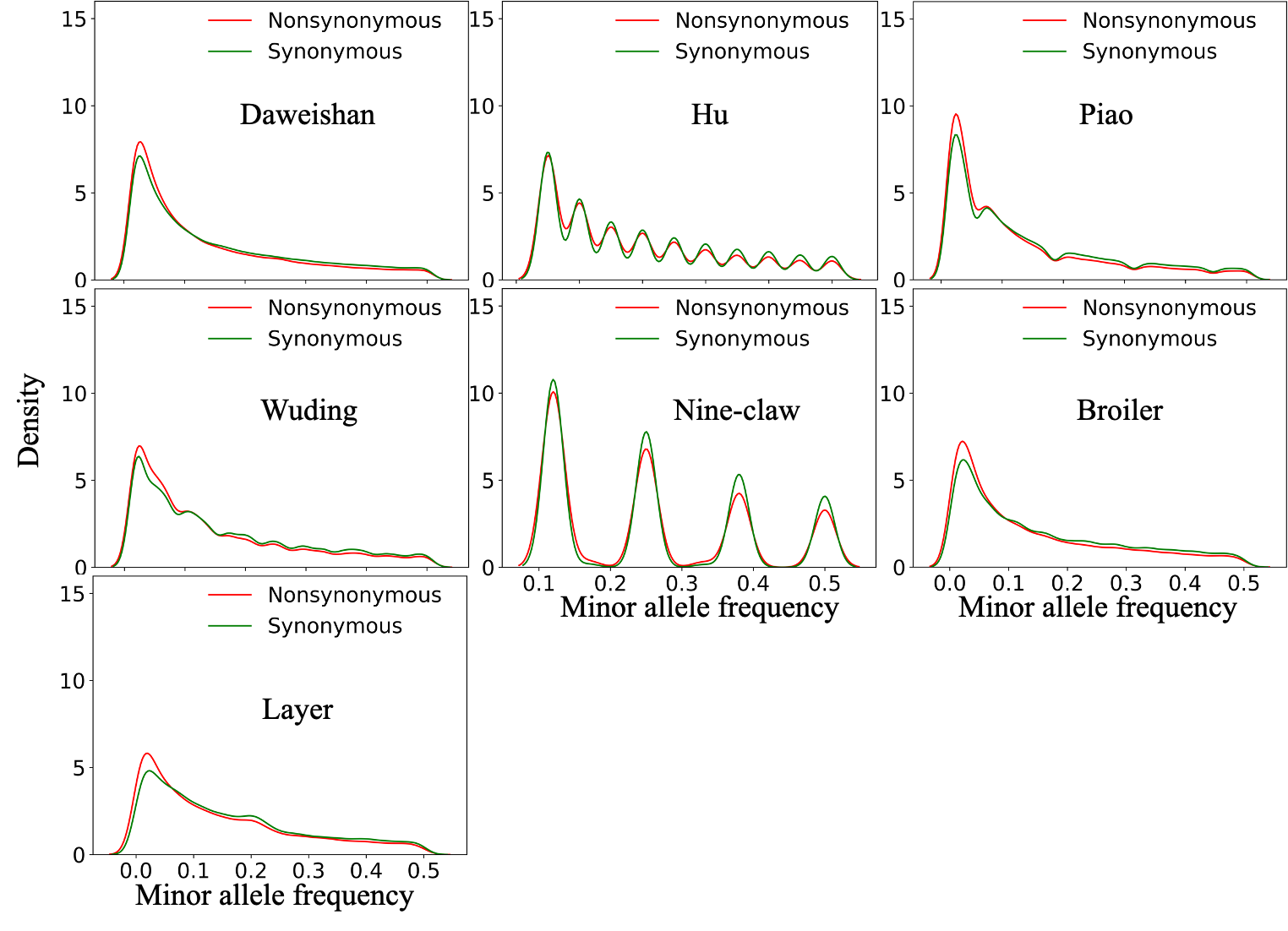


**Figure S1.** Distribution of the minor allele frequency among each chicken breed


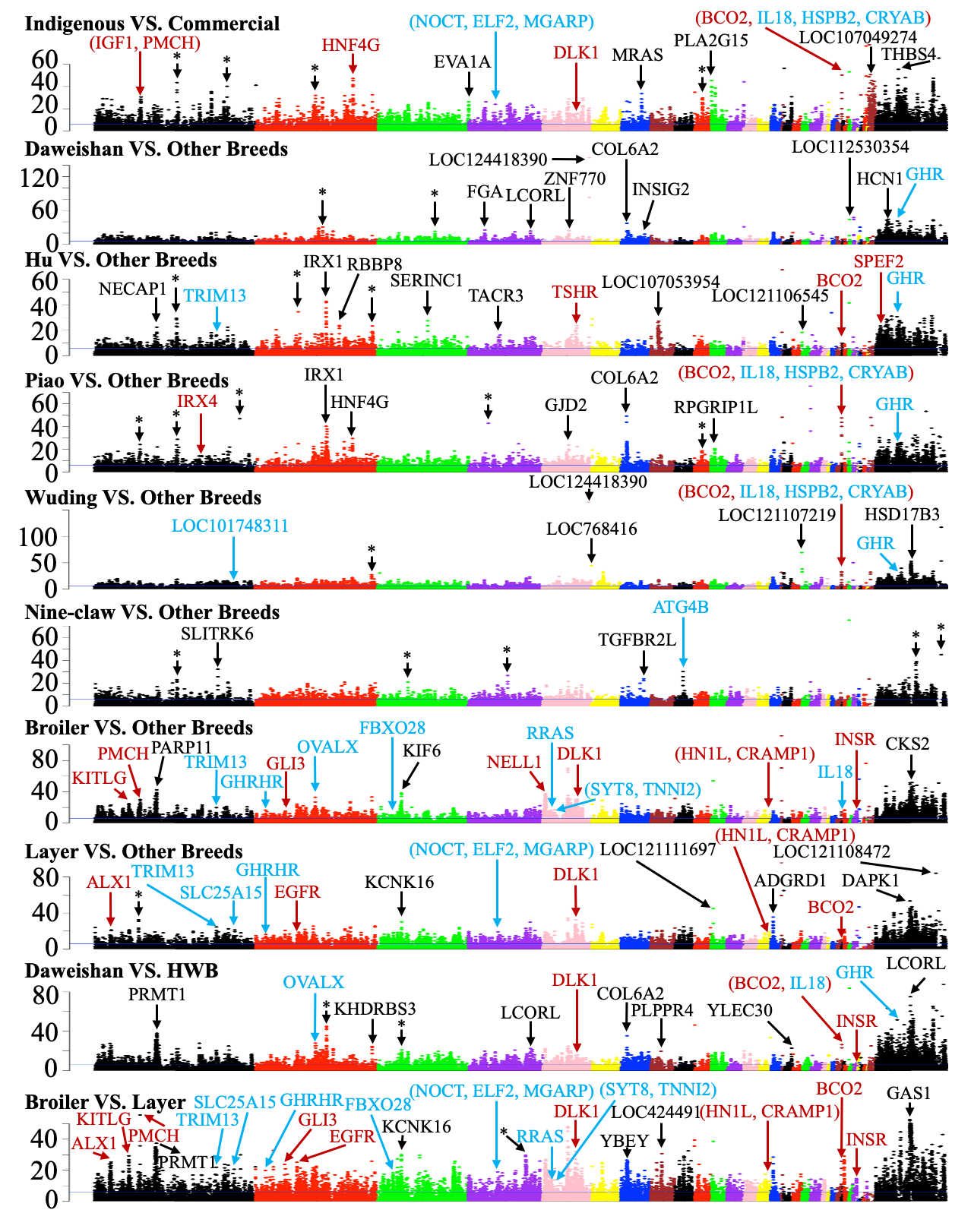


**Figure S2.** Manhattan plots of ${ZF}_{ST}$ values of each window on each chromosome for the indicated comparisons. The blue horizontal line indicates the ${ZF}_{ST}$ cutoff = 6. Examples of genes in significant selective sweep windows are shown in different color. Genes that have been previously reported in selective sweep windows are shown in red, genes in our predicted selective sweep windows potentially related to the specific traits of each chicken breed are shown in blue, and genes in novel selective sweep windows with extremely high ${ZF}_{ST}$ values are shown in black. Asterisk represents selective sweep windows lacking annotated genes.

**
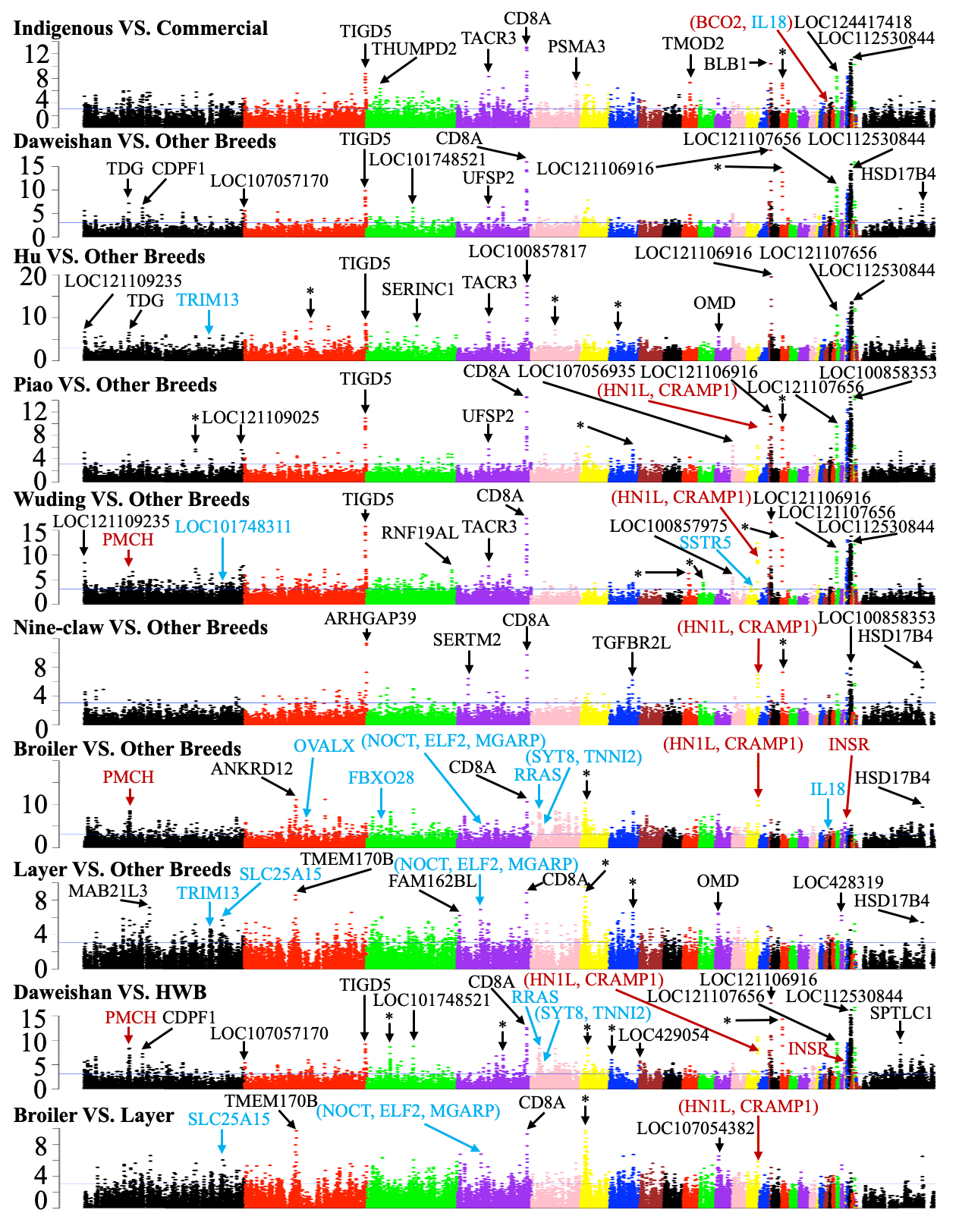
**

**Figure S3.** Manhattan plots of $Z|\pi|$ values of each window on each chromosome for the indicated comparisons. The blue horizontal line indicates the $Z|\pi|$ cutoff = 3.09. Examples of genes in significant selective sweep windows are shown in different color. Genes that have been previously reported in selective sweep windows are shown in red, genes in our predicted selective sweep windows potentially related to the specific traits of each chicken breed are shown in blue, and genes in novel selective sweep windows with extremely high $Z|\pi|$ values are shown in black. Asterisk represents selective sweep windows lacking annotated genes.


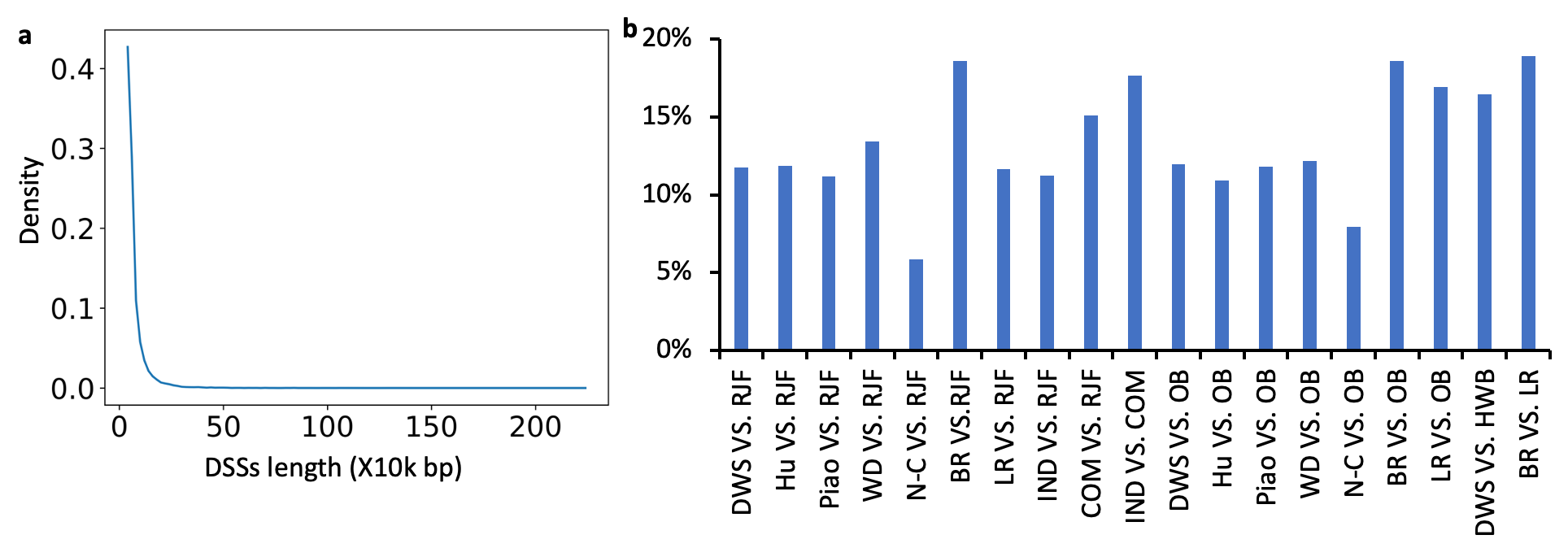


**Figure S4.** Summary of the DSSs lengths. **a.** Distribution of the lengths of the DSSs pooled from the 19 comparisons. **b.** Percentage of the DSSs lengths in each comparison with respect to the length of the reference genome (GRCg7b assembly). Abbreviations: DWS for Daweishan, WD for Wuding, N-C for Nine-claw, BR for Broiler, LR for Layer, IND for indigenous, COM for commercial, OB for Other Breeds and HWB for Hu+Wuding+Broiler.

**Figure S5.** Comparison of genes in DSSs of each indigenous chicken breed shared with those of broilers and layers
