# Supplementary material for: Artificial selection footprints in indigenous and commercial chicken genomes
Source: BMC Genomics. 2024 Apr 30;25:428. doi: 10.1186/s12864-024-10291-5 (PMC11061962; doi:10.1186/s12864-024-10291-5)
Supplement: Supplementary file 2 — Supplementary Material 2. [file 12864_2024_10291_MOESM2_ESM.docx]

**Supplementary Note 1**

**1 β-Actin**

| **Forward primer** | 5′-GAGAAATTGTGCGTGACATCA-3 | 152 |
| --- | --- | --- |
| **Reverse primer** | 5′-CCTGAACCTCTCATTGCCA-3′ |  |

**2 GHR**

|  | Sequence (5'->3') | Tem. strand | Len. | Start | End | Tm | GC% | Self com. | Self 3' com. |
| --- | --- | --- | --- | --- | --- | --- | --- | --- | --- |
| Forward primer | CTGGAGAAGGCCGCATTTTG | Plus | 20 | 112 | 131 | 59.83 | 55.00 | 5.00 | 3.00 |
| Reverse primer | TTGTGGCCACTGCAGAAGAT | Minus | 20 | 284 | 265 | 59.89 | 50.00 | 8.00 | 2.00 |
| Product length | 173 | | | | | | | | |

**3 GHRHR**

|  | Sequence (5'->3') | Tem. strand | Len. | Start | End | Tm | GC% | Self com. | Self 3' com. |
| --- | --- | --- | --- | --- | --- | --- | --- | --- | --- |
| Forward primer | GAGGCTGAATGCCTGGAGAA | Plus | 20 | 173 | 192 | 59.75 | 55.00 | 4.00 | 0.00 |
| Reverse primer | GGGCAAGGTAAGGCAAGAGT | Minus | 20 | 297 | 278 | 59.96 | 55.00 | 2.00 | 1.00 |
| Product length | 125 | | | | | | | | |

**4 OVALX**

|  | Sequence (5'->3') | Tem. strand | Len. | Start | End | Tm | GC% | Self com. | Self 3' com. |
| --- | --- | --- | --- | --- | --- | --- | --- | --- | --- |
| Forward primer | CATCAAGCATTCCCCTGAGTTAGA | Plus | 24 | 1108 | 1131 | 60.38 | 45.83 | 3.00 | 1.00 |
| Reverse primer | ACGGTAAGGGGATTTTGTTTCT | Minus | 22 | 1282 | 1261 | 57.89 | 40.91 | 3.00 | 0.00 |
| Product length | 175 | | | | | | | | |

**5 IGF2BP1**

|  | Sequence (5'->3') | Tem. strand | Len. | Start | End | Tm | GC% | Self com. | Self 3' com. |
| --- | --- | --- | --- | --- | --- | --- | --- | --- | --- |
| Forward primer | GGGAACCTAAACGAGAGCGT | Plus | 20 | 142 | 161 | 59.75 | 55.00 | 3.00 | 2.00 |
| Reverse primer | AAGCATAGCCGGACTTGACC | Minus | 20 | 244 | 225 | 60.11 | 55.00 | 4.00 | 2.00 |
| Product length | 103 | | | | | | | | |

**6 NOCT**

|  | Sequence (5'->3') | Tem. strand | Len. | Start | End | Tm | GC% | Self com. | Self 3' com. |
| --- | --- | --- | --- | --- | --- | --- | --- | --- | --- |
| Forward primer | CACCTGACTGAAGGCGAAGA | Plus | 20 | 1822 | 1841 | 59.68 | 55.00 | 3.00 | 0.00 |
| Reverse primer | CCTACAGACCACGTGTGTGAC | Minus | 21 | 1987 | 1967 | 60.60 | 57.14 | 6.00 | 3.00 |
| Product length | 166 | | | | | | | | |

**7 ELF2**

|  | Sequence (5'->3') | Tem. strand | Len. | Start | End | Tm | GC% | Self com. | Self 3' com. |
| --- | --- | --- | --- | --- | --- | --- | --- | --- | --- |
| Forward primer | ACGCCACCCTCGCCTA | Plus | 16 | 57 | 72 | 59.97 | 68.75 | 2.00 | 2.00 |
| Reverse primer | GGCTTTGGGAGCTGCTATCT | Minus | 20 | 166 | 147 | 59.82 | 55.00 | 5.00 | 2.00 |
| Product length | 110 | | | | | | | | |

**8 MGARP**

|  | Sequence (5'->3') | Tem. strand | Len. | Start | End | Tm | GC% | Self com. | Self 3' com. |
| --- | --- | --- | --- | --- | --- | --- | --- | --- | --- |
| Forward primer | GAAGGCACCGGAGGAAGTTG | Plus | 20 | 768 | 787 | 60.96 | 60.00 | 4.00 | 1.00 |
| Reverse primer | TCTCCGATGAAGAGTCTGAGGT | Minus | 22 | 898 | 877 | 60.03 | 50.00 | 5.00 | 0.00 |
| Product length | 131 | | | | | | | | |

**9 SLC25A15**

|  | Sequence (5'->3') | Tem. strand | Len. | Start | End | Tm | GC% | Self com. | Self 3' com. |
| --- | --- | --- | --- | --- | --- | --- | --- | --- | --- |
| Forward primer | CCTGCTCTGGTAGCCAACAT | Plus | 20 | 566 | 585 | 59.75 | 55.00 | 4.00 | 2.00 |
| Reverse primer | CGGCACTTCACCAGCTCT | Minus | 18 | 759 | 742 | 59.65 | 61.11 | 4.00 | 2.00 |
| Product length | 194 | | | | | | | | |
